# Preferences for HIV prevention strategies among newly arrived Asian-born men who have sex with men living in Australia: A discrete choice experiment

**DOI:** 10.3389/fpubh.2023.1018983

**Published:** 2023-03-13

**Authors:** Megan Ung, Sarah Martin, Fern Terris-Prestholt, Matthew Quaife, Warittha Tieosapjaroen, Tiffany Phillips, David Lee, Eric P. F. Chow, Nick Medland, Benjamin R. Bavinton, Stephen W. Pan, Limin Mao, Jason J. Ong

**Affiliations:** ^1^Department of Infectious Diseases and Microbiology, Concord Hospital, Sydney, NSW, Australia; ^2^Canberra Sexual Health Centre, Canberra, ACT, Australia; ^3^College of Health and Medicine, Australian National University, Canberra, ACT, Australia; ^4^Department of Global Health and Development, London School of Hygiene and Tropical Medicine, London, United Kingdom; ^5^Melbourne Sexual Health Centre, Melbourne, VIC, Australia; ^6^Central Clinical School, Monash University, Melbourne, VIC, Australia; ^7^Kirby Institute, University of New South Wales, Sydney, NSW, Australia; ^8^Department of Health and Environmental Sciences, Xi'an Jiaotong-Liverpool University, Suzhou, China; ^9^University of Liverpool, Liverpool, United Kingdom; ^10^Centre for Social Research and Health, University of Sydney, Sydney, NSW, Australia

**Keywords:** HIV, men who have sex with men, migrants, pre-exposure prophylaxis, Australia, health preference research, discrete choice experiment

## Abstract

The HIV epidemic in Australia is changing with higher risk for HIV among newly-arrived Asian-born men who have sex with men (MSM) compared to Australian-born MSM. We evaluated the preferences for HIV prevention strategies among 286 Asian-born MSM living in Australia for <5 years. A latent class analysis uncovered three classes of respondents who were defined by their preferences: “PrEP” (52%), “Consistent condoms” (31%), and “No strategy” (17%). Compared to the “No strategy” class, men in the “PrEP” class were less likely to be a student or ask their partner for their HIV status. Men in the “Consistent condoms” class were more likely to get information about HIV from online, and less likely to ask their partner for their HIV status. Overall, PrEP was the preferred HIV prevention strategy for newly arrived migrants. Removing structural barriers to access PrEP can accelerate progress toward ending HIV transmission.

## Introduction

Australia has a concentrated HIV epidemic as is the case in many high-income countries. The majority of HIV diagnoses are in men (90%), with the highest acquisition risk in men who have sex with men (MSM) (1). Australia has upheld a successful and sustained approach to reducing the incidence of new HIV diagnoses each year (2). The highest annual cases recorded in Australia were 2,890 cases in 1984 (3). Since then, there has been a steep decline in the number of HIV notifications with 633 new diagnoses in 2020 (1). Australia has supported sequential approaches to HIV prevention, which in turn has contributed to the decline in HIV notifications in the last decade. These preventative methods have ranged from condom use and safer injecting practices to treatment as prevention (TasP), increased frequency of testing and most recently, pre-exposure prophylaxis (PrEP) (4, 5). Australia's healthcare system is such that those who are eligible for Medicare (anyone who is an Australian citizen, permanent resident, or a temporary resident covered by a ministerial order), are able to obtain HIV anti-retroviral therapy and HIV PrEP at a subsidized cost (6). However, people living with HIV who are Medicare-ineligible usually obtain HIV anti-retroviral therapy *via* compassionate access (i.e., provided by pharmaceutical companies), or import from their country of origin or from online pharmacies. Similarly, people who are Medicare-ineligible and desire PrEP at an affordable price often rely on personal importation from international online pharmacies, which is legal in Australia (5).

A greater focus on Asian-born MSM is crucial to end the HIV epidemic in Australia. The number of HIV diagnoses amongst Australian-born MSM has declined by over 44% in the past 5 years, while the number of new HIV diagnoses among overseas-born MSM remained steady between 2016 and 2019 (1). The prevalence of HIV in gay, bisexual and other men who have sex with men living in Australia is 7.3% (7). The prevalence of HIV in Asian born MSM who have recently migrated to Australia is not known. In 2017, research from Melbourne Sexual Health Center (MSHC) indicated that overall, incident HIV infections (infections that occurred within the last year) declined in MSM between 2008 and 2018, but had not declined in newly-arrived (< 5 years) Asian-born MSM (8). Newly-arrived Asian-born MSM are now four times more likely to be diagnosed with incident HIV infection at MSHC, compared to other MSM (9). It is not clear why newly-arrived Asian-born MSM are at a higher risk of incident HIV infection compared to Australian-born MSM despite reporting higher levels of condom use and fewer sexual partners, and being less likely to be diagnosed with other sexually transmitted infections (10). Reduced access to PrEP and HIV testing may explain this divergent trend among newly-arrived Asian-born MSM (11). Another hypothesis is that newly-arrived Asian-born MSM are at higher risk as their sexual networks may be other newly-arrived Asian-born MSM who have a higher rate of undiagnosed infection acquired in countries with lower coverage of HIV testing, treatment and virological suppression than Australia.

Discrete choice experiments (DCEs) have been widely utilized in economics and marketing to assess how much participants value the attributes of a good or service (12). DCE methodology has evolved over time and now is used internationally to measure individual preferences in a wide range of settings, including HIV-related research (13, 14). This preference elicitation method is used to understand which attributes are preferred by observing how participants trade-off between attributes in their decision-making (15). The advantage of using the DCE methodology is by presenting a variety of hypothetical scenarios to participants, the analyst can quantify the relative importance of each attribute or attribute during the decision-making process (16). This study aims to use a DCE methodology to examine preferences for HIV prevention strategies among newly arrived Asian-born MSM.

## Methods

### Development and piloting of choice tasks, attributes, and levels

**Figure 1:**
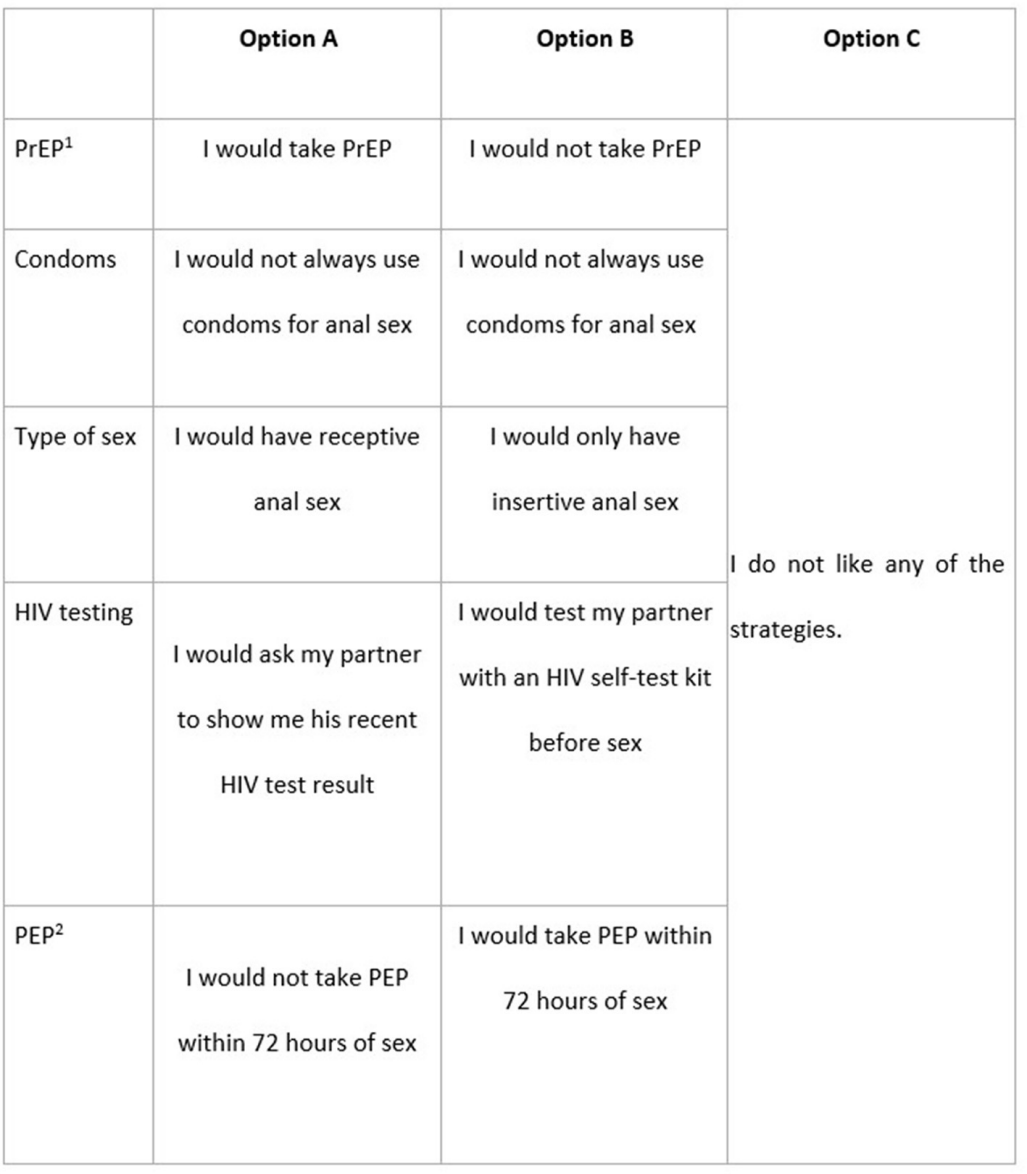
Example choice set. Imagine you are going to have sex with someone where you are not completely certain he is HIV-negative. What strategy would you prefer to protect yourself against HIV? ^1^PrEP, Pre-exposure prophylaxis; ^2^PEP, Post-exposure prophylaxis.

For the formative step of the DCE, we conducted semi-structured in-depth interviews with 24 newly-arrived Asian-born MSM between May to December 2019 to understand their experience of HIV testing and type of prevention strategies (17, 18). We found most men relied on condoms for HIV prevention but many indicated an interest in accessing PrEP. Using data from the first 14 interviews, we presented an example choice set for the DCE survey to the final 10 interviewees to obtain their feedback on the comprehensibility of the choice scenarios and choice sets and whether there were any missing HIV prevention strategies. Final DCE survey attributes and levels were determined (Table 1). The participants were asked to choose between two unlabeled alternatives or an opt-out alternative (Figure 1). The choice scenario was presented to all participants: “Imagine you are going to have sex with someone where you are not completely certain he is HIV-negative. What strategy would you prefer to protect yourself against HIV?”

**Table 1:** Attributes and levels of the discrete choice experiment.

**Attributes**	**Levels**
PrEP^1^	I would take PrEP I would not take PrEP I would check if my partner is on PrEP
Condoms	I would always use condoms for anal sex I would not always use condoms for anal sex
Type of sex	I would only have oral sex I would only have insertive anal sex I would have receptive anal sex
HIV testing	I would not talk about HIV testing with him I would test my partner with an HIV self-test kit before sex I would ask my partner to show me his recent HIV test result
PEP^2^	I would take PEP within 72 h of sex I would not take PEP within 72 h of sex

1 PrEP, Pre-exposure prophylaxis.

2 PEP, Post-exposure prophylaxis.

### Survey instrument

The DCE survey contained questions about participants' sociodemographic characteristics, including their age, education level, English fluency, sexual identity, connectedness to the gay community in Australia and their home country, use of HIV prevention strategies and sexual behaviors in the preceding 6 months. We used NGENE Software (Version 1.2.1, Choicemetrics, USA) to construct a D-efficient experimental design to maximize the information from each choice set. The experimental design contained 18 choice sets which we blocked into two so each participant would only see nine choice sets. We translated the survey into Thai, Simplified Chinese and Vietnamese with a professional translation service. The translated versions were sent to at least five MSM fluent in these languages to check the appropriateness of terminologies and comprehensibility of the survey.

### Study recruitment

The online survey was constructed using SurveyEngine. We recruited participants through online advertisements to users of the gay dating apps (Blued and Grindr), postcards (with QR code) of clients attending Melbourne Sexual Health Centre and Canberra Sexual Health Centre, social media (Facebook groups, Twitter), community-based organization email lists and the University of Melbourne student online portal from February 2020 to February 2021. Potential participants were shown an advertisement that links to an online survey for those interested in the survey. Those who were eligible to participate were MSM, not diagnosed with HIV, over 18 years old, born in Asia (South Asia, East Asia, South-East Asia), and arrived in Australia <5 years prior. Each participant read an online consent form and consented to participate by opening the survey. The minimum sample size was calculated using a standard of at least 100 respondents needed to model preference data (19).

### Statistical analysis

We used descriptive statistics to summarize the sociodemographic characteristics of study participants. We used random parameters logit (RPL) models to analyze the choice data. The models were estimated using a maximum likelihood approach with 1000 Halton draws. All parameters were set to have an underlying normal distribution. We calculated the log-likelihood and Akaike information criteria to assess model fit. Qualitative variables were effect coded (20). The coefficients represent the relative strength of preference for the attribute level (higher magnitude of a positive coefficient represents an attribute that is more preferred whilst a higher magnitude of a negative coefficient represents an attribute that is less preferred). If the standard deviation (SD) for an attribute level is statistically significant, this indicates an unobserved heterogeneous spread of preferences for this attribute level. The attribute with the largest range in its coefficients indicates the relative importance of that attribute (21). To explore the heterogeneity of preferences and the popularity of various HIV prevention strategies, we conducted a latent class analysis (LCA) to identify clustering of similar preferences for HIV prevention strategies among recently-arrived Asian-born MSM. The LCA is a statistical procedure that allows the identification of underlying and unobservable latent variables using observed indicators, in order to determine subgroups for targeting interventions (22). All model estimations were performed using NLOGIT 6 (version 6, Econometric Software Inc., USA). We conducted a supplementary analysis in STATA (version 17.0, Statacorp) using logistic regression models to compare the sociodemographic characteristics of men who had the highest probability of being classified as having “no strategy”.

## Results

In total, 1,475 individuals clicked on the initial survey, and 286 (19%) completed the survey. Their mean age was 29.2 (SD 6.8) and mean duration living in Australia was 2.8 years (SD 1.4). The mean number of regular male sex partners in the last 6 months was 2.0 (SD 4.0) and the average number of casual male sex partners in the last 6 months was 6.0 (SD 11.5). Most (94%) had tested for HIV within 1 year and a quarter were taking PrEP (Table 2).

**Table 2:** Sociodemographic characteristics of the study population (*N* = 286).

**Sexual identity**	***n***	**%**
Gay/homosexual	233	81.5
Bisexual	46	16.1
Straight/heterosexual	1	0.3
Queer	3	1.0
Pansexual	1	0.3
**Highest education level**		
Up to high school (including secondary or primary school)	24	8.4
Tertiary diploma or trade certificate (TAFE^1^, vocational training/private college)	40	14.0
English college	3	1.0
Undergraduate university degree	111	40.9
Postgraduate university degree	106	38.8
Other	2	0.7
**Ever tested for HIV**	272	95.1
**Time since last HIV test (*****N*** = **272)**		
Within the last year	255	93.8
1–2 years ago	14	5.1
More than 2 years ago	3	1.1
**Strategy used at last sex** ^i^		
Using a condom for anal sex	103	36.0
PrEP^2^	71	24.8
Asking if my partner was on PrEP before I had sex	69	24.1
Asking my partner to show me his most recent HIV test result	47	16.4
Only having oral sex	45	15.7
Only having insertive anal sex (top role)	32	11.2
Taking post-exposure prophylaxis within 72 h of having sex	25	8.7
No strategy	11	3.8
Testing my partner for HIV using a self-test kit before sex	6	2.1
Always use condoms with regular partners in the last 6 months (*N* = 171)	40	23.4
Always use condoms with casual partners in the last 6 months (*N* = 180)	70	38.9

1 TAFE, Technical and Further Education.

2 PrEP, Pre-exposure Prophylaxis.

i Participants can choose more than one strategy.

Generally, when faced with the possibility of having sex with a partner of unknown HIV status, men preferred using PrEP themselves or ensuring their partner took PrEP, and disliked the option with no PrEP (Table 3). Men preferred always using condoms compared to inconsistent condom use. Men preferred checking their partner's HIV test results and disliked no discussion about HIV status. The most important to least important HIV prevention strategy was PrEP (34.8%), condom use (32.3%), post-exposure prophylaxis (PEP) (20.1%), asking their sexual partners for their latest HIV test result (10.0%), and seropositioning (i.e., selectively practicing the type of sex based on partner's HIV status) (2.7%). There was significant preference heterogeneity noted for PrEP strategies, condoms, seropositioning and PEP.

**Table 3:** Preferences for HIV prevention strategies among overseas born MSM (*N* = 286), random parameters logit model.

**Attributes**	**Coefficient**	**Standard error**	**Standard deviation**	**Standard error**
**PrEP** ^1^				
No PrEP	−0.69^***^	0.09	0.73^***^	0.15
PrEP	0.45^***^	0.08	0.62^***^	0.11
Partner PrEP	0.24^***^	0.07	0.38^***^	0.10
**Condoms**				
Inconsistent	−0.53^***^	0.07	0.70^***^	0.08
Always	0.53^***^	0.07	0.70^***^	0.08
**Type of sex**				
Oral sex only	−0.05	0.11	0.71^***^	0.19
Insertive anal sex	0.04	0.07	0.53^***^	0.12
Receptive anal sex	0.01	0.07	0.47^***^	0.12
**HIV test**				
No discussion	−0.12^**^	0.07	0.31	0.21
HIVST^2^ before sex	−0.09	0.07	0.17	0.17
Partner result	0.21^***^	0.07	0.26	0.14
**PEP** ^3^				
No PEP	−0.33	0.46	3.52^***^	0.32
PEP	0.33	0.46	3.52^***^	0.32
Opt out	−0.91	0.47		

AIC/N = 2.012, Log-likelihood function = −2,182.7.

** p-value < 0.05.

*** p-value < 0.01.

1 PrEP, Pre-exposure prophylaxis.

2 HIVST, HIV self-testing.

3 PEP, Post-exposure prophylaxis.

For the latent class analysis (Table 4), we found three classes. The largest class (PrEP, 52%) was characterized by men who preferred PrEP (ß = 0.38) more than twice as much as consistent condom use (ß = 0.15). Asking partner's latest HIV test result (ß = 0.15) was equally preferred to consistent condom use. People in this class were less likely to be students or to ask partner's latest HIV test result. The next largest class (Condoms, 31%) preferred consistent condom use (ß = 0.91) five times more than taking PrEP themselves (ß = 0.18). They had a greater preference for their partner to be on PrEP (ß = 0.29) rather than themselves (ß = 0.18). They equally preferred asking about their partner's latest HIV test result (ß = 0.28) as the partner taking PrEP (ß = 0.29). People in this class were more likely to be seek HIV information online, used condoms at their last sex, and less likely to ask partners about their latest HIV test result. The smallest class (No strategy, 17%) were most likely to opt out (ß = 3.00), i.e., they did not prefer the strategies presented in the choice set. They were more likely to ask their partners about their latest HIV test. To further unpack the characteristics of this class for whom HIV prevention did not appear a highly salient issue, we analyzed their characteristics. Besides having greater odds of being a student (OR 1.84, 95% CI: 0.99–3.45), we did not find any other statistically significant factors associated with the “No strategy” class (Supplementary Table 1).

**Table 4:** Latent class analysis for HIV prevention strategies.

**Attributes**	**PrEP**	**Condoms, partner PrEP, seropositioning**	**No strategy**
	**Class size: 51.7%**	**31.4%**	**16.9%**
PrEP^1^	Coefficient (standard error)	Coefficient (standard error)	Coefficient (standard error)
No PrEP	−0.46 (0.05)^***^	−0.47 (0.11)^***^	−0.51 (0.35)
PrEP	0.38 (0.06)^***^	0.18 (0.11)^*^	0.17 (0.30)
Partner taking PrEP	0.08 (0.05)	0.29 (0.10)^***^	0.34 (0.28)
**Condoms**			
Inconsistent	−0.15 (0.04)^***^	−0.91 (0.10)^***^	−0.08 (0.24)
Consistent	0.15 (0.04)^***^	0.91 (0.10)^***^	0.08 (0.24)
**Type of sex**			
Oral sex only	−0.08 (0.08)	0.20 (0.15)	−0.22 (0.43)
Insertive anal sex	0.04 (0.06)	−0.07 (0.11)	−0.23 (0.34)
Receptive anal sex	0.04 (0.05)	−0.13 (0.11)	0.45 (0.30)
**HIV test**			
No discussion	−0.10 (0.06)	−0.15 (0.11)	−0.39 (0.44)
HIVST^2^ before sex	−0.05 (0.05)	−0.13 (0.11)	0.31 (0.34)
Partner result	0.15 (0.06)^***^	0.28 (0.10)^***^	0.08 (0.31)
**PEP** ^3^			
No PEP	−0.08 (0.14)	−0.08 (0.17)	−0.29 (0.31)
PEP	0.08 (0.14)	0.08 (0.17)	0.29 (0.31)
Opt out	−3.07 (0.31)^***^	0.30 (0.19)	3.00 (0.36)^***^
Class membership prediction	Theta (standard error)	Theta (standard error)	
Online	0.30 (0.23)	0.68 (0.30)^**^	Reference
Used condoms with last sex	−0.11 (0.25)	0.61 (0.29)^**^	Reference
Asked partners about their HIV result at last sex	−0.71 (0.25)^***^	−0.61 (0.28)^**^	Reference
Students	−0.40 (0.23)^*^	−0.20 (0.25)	Reference

AIC/N = 1.679, LL function = −1,798.97.

* p-value < 0.10.

** p-value < 0.05.

*** p-value < 0.01.

1 PrEP, pre-exposure prophylaxis.

2 HIVST, HIV self-testing.

3 PEP, post-exposure prophylaxis.

## Discussion

Our study measured preferences for HIV prevention strategies amongst Asian-born MSM living in Australia. We add to the sparse literature for newly-arrived Asian-born MSM who have a higher risk for HIV compared with their Australian-born counterparts (9). We found that Asian-born MSM strongly preferred PrEP as a HIV preventive strategy, followed closely by consistent use of condoms. There was heterogeneity in preferences for HIV prevention strategies according to where people sought information about HIV, strategy used at last sex and whether they were students.

Overall, PrEP was a strongly preferred HIV preventative method in our study. It was noted that those who prefer PrEP strongly were also less likely to consistently use condoms. This finding is similar to a latent class analysis of MSM living in Montreal, whereby MSM who were PrEP users also had a low probability of using condoms consistently (23). However, in the Australian context, there is a discrepancy between the preference to use PrEP and the ease of accessing PrEP services for those who are newly-arrived in Australia. Newly-arrived MSM who are not familiar with navigating the Australian health system may not be aware of current, more affordable options to access PrEP and may be unable to pay for accumulative costs for a doctor's consultation (~$70), HIV and sexually transmitted infections (STIs) testing (~$200) and buying an unsubsidized PrEP script (30 pills cost ~$70) (24, 25). These structural barriers can deter newly-arrived MSM from seeking services including HIV testing and PrEP (26). Other existing barriers for newly-arrived MSM in Australia to access PrEP also include social and cultural barriers. Asian-born MSM may have originated from countries where stigma and discrimination surrounding same-sex attraction and HIV is highly prevalent (27). This may be in part why our study also found newly arrived MSM who were students were less likely to prefer using PrEP and more likely to be in the “no strategy” latent class. Approximately 350,000 international students from Asia come to Australia each year for higher education (28). International students living in Australia report concerns about hidden costs for accessing health services (27). In addition, a recent Australian study found most of the 111 recently arrived Asian MSM diagnosed with HIV did not have access to Medicare and were international students holding temporary visas (10). Currently, HIV testing is not mandatory for people applying for a temporary visa to stay in Australia (29). A possible solution to mitigate the incidence of HIV in Australia is to offer free HIV testing to people applying for temporary visas in Australia and affordable treatment where necessary. Furthermore, a focus on eliminating the barriers to preventative healthcare for recently arrived Asian-born MSM (particularly students) is a critical factor in reducing the incidence of HIV in this subpopulation.

About a third of men strongly preferred consistent use of condoms as a HIV preventive method but were less likely to prefer PrEP. Consistent condom use is a key HIV prevention strategy, as well as for reducing STIs acquisition. Lack of consistent condom use places at-risk groups at a higher risk of acquiring HIV if they are not relying on PrEP or undetectable viral load (30). Nonetheless, consistent condom use may be difficult to sustain, particularly given the falling rates of consistent use among MSM with the increasing uptake of PrEP in the recent years. Condomless anal sex with casual partners amongst MSM living in Australia has increased substantially from 53.7% in 2017 to 65.2% in 2021 (31). We found that those who sought information about HIV online were more likely to choose condom use as their HIV prevention strategy. Thus, there may be an opportunity to increase awareness about PrEP using eHealth platforms. eHealth platforms could eliminate fears of judgment and non-confidential clinical settings and allow for more convenient access to PrEP (32). For example, in Thailand, “Adam's Love” is a leading online outreach platform for PrEP awareness and counseling among the Thai MSM and transgender population and has facilitated recruitment and enrolment into PrEP studies (33). However, there is limited evidence on available eHealth platforms for PrEP access in other Asia-Pacific countries. Offering an online health service with telemedicine, counseling, prevention information, HIV testing and PrEP delivery may be worth exploring to target the newly arrived Asian MSM in Australia who find it difficult to navigate the Australian health system.

Worryingly, we found that a significant minority (17%) were categorized as having “no strategy”. It is not clear from our survey the reasons for not preferring any HIV prevention strategy. This might relate to factors not measured in our study such as low perception of risk, low health literacy, competing priorities in moving to a new country or genuine dislike of available HIV prevention strategies. Factors that have been associated with the non-uptake of PrEP in Australia among MSM (both Australian-born and Asian-born) include younger age, being less socially engaged with gay community, low perceived and/or actual risk of HIV and concerns of medication-related side effects of PrEP and taking daily medication (34, 35). A lack of Medicare coverage has also been independently associated with not using PrEP despite a willingness to use it (36). Qualitative research from 24 newly-arrived Asian-born MSM reported significant barriers including shame, stigma and fear related to HIV and their sexual identity remained despite living in a more liberal society (17, 18). More implementation research is needed to understand how to address the barriers of newly arrived Asian-born MSM to using effective HIV prevention strategies.

The strength of our study is that we quantitatively measured the preferences for HIV prevention strategies among Asian-born MSM to better understand their current preferences. We also uncovered subpopulations within this population with unique preferences to enable more targeted approaches to mitigate their risk of HIV acquisition. Our analysis has limitations. Firstly, there is potential for selection bias as we recruited mainly from sexual health clinics. These men may have higher health literacy than those not attending a sexual health clinic and may already be aware of HIV prevention strategies. Finding marginalized populations to ensure they have access to information and healthcare to inform their choice of HIV prevention strategies remains an ongoing challenge (37). Similarly, ensuring a representative sample of Asian-born MSM may not be possible, given the diversity of MSM from different countries in Asia. Future studies should explore the impact of diverse cultures and countries of origin on HIV prevention strategies. Secondly, there is potential for hypothetical bias inherent in stated choice surveys. However, recent reviews have demonstrated that health-related DCEs have adequate external validity (15, 38) and remain the best method to use when revealed preference data are not practical or possible to obtain. Third, our response rate was relatively low (19%), which may reflect the challenges in conducting research regarding sensitive issues such as HIV and sexual health.

## Conclusions

In the context of higher risk of HIV acquisition among newly-arrived Asian-born MSM compared to Australian-born MSM, our study highlighted that overall, PrEP is strongly preferred as an HIV prevention strategy despite the current difficulties to access PrEP for this population.

## Data availability statement

The raw data supporting the conclusions of this article will be made available by the authors, without undue reservation.

## Ethics statement

Ethics review and approval was granted by the Alfred Human Research Ethics Committee (222/19). Informed consent was obtained from the participants for the participation in this study.

## Author contributions

JO, NM, FT-P, MQ, and SP contributed to the study conception and design. Material preparation, data collection, and analysis were performed by MU, SM, FT-P, MQ, DL, EC, SP, and JO. The first draft of the manuscript was written by MU and JO. All authors commented on previous versions of the manuscript and read and approved the final manuscript.
