# Supplementary material for: Preferences for HIV prevention strategies among newly arrived Asian-born men who have sex with men living in Australia: A discrete choice experiment
Source: Front Public Health. 2023 Mar 13;11:1018983. doi: 10.3389/fpubh.2023.1018983 (PMC10040803; doi:10.3389/fpubh.2023.1018983)
Supplement: Supplementary file 1 [file Table_1.DOCX]

**Supplementary Table 1 Logistic regression comparing ‘No strategy’ class with the remaining study population**

| **Characteristic** | **OR (95% CI)** | **p value** |
| --- | --- | --- |
| **Age (per year increase)** | 0.97 (0.92-1.02) | 0.265 |
| **Sexual identity** |  |  |
| Gay / Homosexual | 1 |  |
| Bisexual | 1.25 (0.56-2.80) | 0.590 |
| Straight/Heterosexual | - |  |
| Queer | 2.57 (0.23-29.01) | 0.446 |
| Pansexual | - |  |
| **Highest education level** |  |  |
| Up to high school (including secondary or primary school) | 1 |  |
| Tertiary diploma or trade certificate (TAFE, vocational training/private college) | 1.29 (0.34-4.86) | 0.706 |
| English College | - |  |
| Undergraduate university degree | 1.11 (0.34-3.61) | 0.861 |
| Postgraduate university degree | 0.70 (0.21-2.37) | 0.565 |
| Other | - | - |
| **Ever tested for HIV** |  |  |
| No | 1 |  |
| Yes | 2.59 (0.75-8.98) | 0.133 |
| **Time since last HIV test** *(N=272)* |  |  |
| Within the last year | 1 |  |
| 1-2 years ago | 2.15 (0.64-7.19) | 0.214 |
| More than 2 years ago | - |  |
| **Strategy used at last sex^1^** |  |  |
| Using a condom for anal sex | 0.80 (0.40-1.59) | 0.521 |
| PrEP | 0.75 (0.35-1.60) | 0.460 |
| Asking if my partner was on PrEP before I had sex | 0.91 (0.44-1.92) | 0.814 |
| Asking my partner to show me his most recent HIV test result | 3.14 (1.50-6.56) | 0.002 |
| Only having oral sex | 1.15 (0.50-2.62) | 0.742 |
| Only having insertive anal sex (top role) | 0.60 (0.20-1.81) | 0.363 |
| Taking post-exposure prophylaxis within 72 hours of having sex | 1.48 (0.55-3.97) | 0.437 |
| No strategy | 0.43 (0.05-3.48) | 0.431 |
| Testing my partner for HIV using a self-test kit before sex | - |  |
| **Always use condoms with regular partners in the last 6 months (*N*=171)** | 1.54 (0.68-3.49) | 0.300 |
| **Always use condoms with casual partners in the last 6 months (*N*=180)** | 0.78 (0.37-1.66) | 0.521 |
| **Student** |  |  |
| No | 1 |  |
| Yes | 1.84 (0.99-3.45) | 0.055 |
| **Fulltime worker** |  |  |
| No | 1 |  |
| Yes | 0.50 (0.24-1.06) | 0.072 |
| **Sex in the last 6 months** |  |  |
| No | 1 |  |
| Yes | 0.46 (0.15-1.36) | 0.160 |
| **Group sex in the last 6 months** |  |  |
| No | 1 |  |
| Yes | 0.51 (0.21-1.23) | 0.131 |
| **Sex with sex worker in the last 6 months** |  |  |
| No | 1 |  |
| Yes | 1.12 (0.73-1.71) | 0.615 |
| **Worked as a sex worker in the last 6 months** |  |  |
| No | 1 |  |
| Yes | 0.92 (0.63-1.36) | 0.691 |
| **Easy to understand choices** |  |  |
| Not easy or hard / Hard / Very Hard | 1 |  |
| Easy or very easy | 0.77 (0.40-1.47) | 0.424 |
| **Paid attention to all strategies before made choice** |  |  |
| No | 1 |  |
| Yes | 1.65 (0.81-3.38) | 0.167 |
| **Time of completion of survey** |  |  |
| >10 minutes | 1 |  |
| <10 minutes | 0.90 (0.19-4.18) | 0.890 |
